# The association of comorbidity measures and mortality in geriatric rehabilitation inpatients by cancer status: RESORT

**DOI:** 10.1007/s00520-020-05967-z

**Published:** 2021-01-18

**Authors:** Cheuk Huen Chan, Claire Maddison, Esmee M. Reijnierse, Wen Kwang Lim, Andrea B. Maier

**Affiliations:** 1 Department of Medicine and Aged Care, @AgeMelbourne, The Royal Melbourne Hospital, The University of Melbourne, Parkville, Victoria Australia; 2 Department of Human Movement Sciences, @AgeAmsterdam, Faculty of Behavioural and Movement Sciences, Amsterdam Movement Sciences, Vrije Universiteit Amsterdam, van der Boechorststraat 7, 1081 BT Amsterdam, The Netherlands

**Keywords:** Geriatrics, Cancer, Comorbidity, Mortality, Aged

## Abstract

**Background:**

Multimorbidity is highly prevalent in older adults, both those with and without cancer, and is associated with an increased risk of mortality. The aim of this study was to investigate if multimorbidity measures in geriatric rehabilitation inpatients differ in their association with mortality, dependent on a diagnosis of cancer.

**Methods:**

REStORing health of acutely unwell adulTs (RESORT) is an ongoing longitudinal inception cohort of geriatric rehabilitation inpatients. Comorbidity was measured at admission using the Charlson Comorbidity Index (CCI), age-adjusted CCI (CCI-A), Cumulative Illness Rating Scale–Geriatrics (CIRS-G) and the CIRS-G severity index. Patients were allocated to a cancer status group (no cancer, history of cancer, or active cancer). The association of comorbidity indices with mortality was analyzed using Cox regression analyses.

**Results:**

Of the 693 patients (mean age 82.2 ± 7.5 years), 523 (75.4%) had no history of cancer, 96 (13.9%) past cancer, and 74 (10.7%) active cancer. Three months post-discharge, patients with active cancer had a higher mortality risk compared to patients with no cancer (HR = 3.57, 95% CI 2.03–6.23). CCI and CCI-A scores were significantly associated with higher mortality risk in all cancer status groups.

**Conclusion:**

In geriatric rehabilitation patients, incremental CCI and CCI-A scores were associated with higher mortality in all three cancer status groups. However, patients with active cancer had a significantly higher 3-month mortality compared to those with no or past cancer, and this is likely determined by the advanced nature of the malignancies in this group.

**Supplementary Information:**

The online version contains supplementary material available at 10.1007/s00520-020-05967-z.

## Introduction

Cancer is a leading cause of disease burden and mortality worldwide, and the majority of diagnoses are made in individuals aged 65 years or older [1, 2]. Multimorbidity, the concurrent presence of two or more medical conditions in an individual [3], increases in prevalence with age in both the general [3] and oncological populations [4]. It may be measured using a number of validated assessment tools, the Charlson Comorbidity Index (CCI) and Cumulative Illness Rating Scale–Geriatric (CIRS-G) version being two of the most commonly utilized [5].

The presence and severity of multimorbidity influences aspects of medical care from diagnosis to treatment decision-making, deliverability, and tolerability. Higher multimorbidity scores predict greater mortality following discharge from internal and geriatric medicine wards, particularly over longer follow-up periods [5]. However, to our knowledge, there are currently no data describing their relationship with mortality in geriatric rehabilitation patients. In patients with cancer, higher comorbidity scores are associated with higher mortality risk [6], but this varies according to cancer type and stage [7], having less impact in those with advanced or rapidly proliferating cancers [7]. In the context of higher life expectancy and the expanding treatment options for older patients diagnosed with cancer, it is likely that an increasing number of older cancer patients with comorbidities will be admitted to geriatric rehabilitation wards. Understanding their ability to benefit from geriatric rehabilitation, and how that differs from patients without cancer, is critical to ensure appropriate care and resource utilization.

The aim of this study was to assess the association of multimorbidity, measured by the CCI and CIRS-G, with mortality 3 months post-discharge in geriatric rehabilitation patients with no, past, and active cancer in their medical history.

## Methods

### Study design and setting

This analysis is based on the first wave of patients participating in the REStORing health of acutely unwell adulTs (RESORT) study between 15 October 2017 and 31 August 2018, an ongoing prospective, longitudinal, observational inception cohort. Patients on the geriatric rehabilitation wards of the Royal Melbourne Hospital, Melbourne, Australia, completed a standardized comprehensive geriatric assessment (CGA) at both admission and discharge. Written informed consent was obtained by the patient or a nominated proxy. Patients were excluded if they were receiving palliative care at admission, were transferred to acute care prior to consenting to the study, or lacked both the capacity to provide informed consent and a nominated proxy.

### Standardized comprehensive geriatric assessment

This multi-disciplinary assessment utilizing validated tools included evaluation of each patients biological, medical, physical, cognitive, psychological, and social functioning [8]. A patient and carer questionnaire collected demographic and personal information. Primary admission reasons were categorized by diagnosis into the following categories: musculoskeletal, neurological, infection, cardiovascular, gastrointestinal and respiratory, and other (including urology, metabolic, psychiatric, vascular, hematologic, and ophthalmological). Cognitive impairment was assessed by physicians and defined as being present if it was endorsed on either the CCI or CIRS-G; dementia or mild cognitive impairment/minor neurocognitive disorder was listed in the discharge summary as a diagnosis; or in the presence of any of a standardized Mini-Mental State Examination (sMMSE) [9] score of < 24 points, a Montreal Cognitive Assessment (MoCA) [10] score < 26 points, or a Rowland Universal Dementia Assessment Scale (RUDAS) [11] score < 23 points. Activities of daily living (ADLs) and instrumental activities of daily living (iADLs) were assessed by occupational therapists using the Katz Index [12] and Lawton Brody Scale [13].

### Cancer status

Patients were assigned to one of three cancer status groups. (1) No cancer: no documented history of melanoma or a solid organ or hematological malignancy. Squamous cell carcinomas and basal cell carcinomas of the skin and all typically non-malignant tumors such as meningiomas and adenomas were included in the no cancer group due to their typically benign influence on prognosis. (2) Past cancer: those with a documented history of melanoma or a solid organ or hematological malignancy that had been treated with curative intent and with no evidence of recurrence on clinical, pathological, or radiological grounds at the time of admission to geriatric rehabilitation. Patients may be on ongoing adjuvant hormone therapy for resected early breast cancer. (3) Active cancer: those with current evidence of melanoma or a solid organ or hematological malignancy on clinical, pathological, or radiological grounds that had either not yet been treated, was being treated, including those being treated with palliative intent, or was not being treated at all.

### Multimorbidity measures

Multimorbidity was documented by the treating physicians at the time of admission according to the Charlson Comorbidity Index (CCI), age-adjusted Charlson Comorbidity Index (CCI-A), the Cumulative Illness Rating Scale–Geriatrics (CIRS-G) version, and the CIRS-G severity index. The CCI includes 19 prespecified conditions that are each assigned a score of 1, 2, 3, or 6 based on their relative risk of death [14]. These scores are summed to produce a final score that may range from 0 to 32. The CCI-A was calculated from the CCI, by adding a single point for each 10 years over the age 40 years [15]. The CIRS-G is an organ-system based rating scale, with the most severe condition occurring in each of 14 organ systems assigned a severity score from 0 (no problem) to 4 (extremely severe), resulting in a final score ranging from 0 to 56 [16]. The CIRS-G severity index was calculated by dividing the total CIRS-G score by the number of organ systems endorsed in the CIRS-G and provides an estimate of the overall severity of dysfunction.

### Mortality

Mortality was captured 3 months post-discharge and determined from hospital records and a phone call made to all participants (or their nominated representative). Time to death was calculated from the date of admission to the date of death or censored at the last date of follow-up.

### Statistical analysis

Continuous data with a normal distribution are presented as mean ± SD and continuous data that were not normally distributed were presented as medians and IQR. Categorical data were presented as counts (frequency) and percentage (%). Patient characteristics were compared between the no, past, and active cancer groups using *χ*^2^ test, Fisher’s exact test, and Kruskal–Wallis tests. The CCI, CCI-A, CIRS-G, and CIRS-G severity scores were assessed as a continuous variable.

Survival analysis of the three cancer status groups was performed by the Kaplan–Meier method. The association between cancer status and mortality, and between comorbidity index score and mortality stratified by cancer status, were assessed with Cox proportional-hazard regression models expressed as HRs and 95% CI. The crude model was presented and an adjusted model for age and sex (except for the CCI-A). The level of statistical significance was set to *p* < 0.05. All statistical analyses were performed using the Statistical Package for the Social Sciences (IBM SPSS Advanced Statistics 25.0, Armonk, NY: IBM Corp).

## Results

The mean age of 693 included patients was 82.2 ± 7.5 years and 392 (56.6%) were female. Of the 693 patients, 523 (75.4%) had no history of cancer, 96 (13.9%) past cancer, and 74 (10.7%) had active cancer. Table 1 shows the patient characteristics for each of the three cancer status groups.

**Table 1:** Patient characteristics, stratified by cancer status

Characteristics	Total (*N* = 693)		Cancer status					
			No cancer (*n* = 523)		Past cancer (*n* = 96)		Active cancer (*n* = 74)	
Demographics								
Age, years	693	82.2 ± 7.51	523	82.3 ± 7.80	96	*82.4 ± 7.90*	74	81.8 ± 8.66
Females	693	392 (56.6)	523	318 (60.8)	96	*45 (46.9)*	74	29 (39.2)
Australian-born	683	301 (44.1)	517	227 (43.9)	95	45 (47.4)	71	29 (40.8)
Multimorbidity								
CCI, score	693	2 [1–4]	523	2 [1–3]	96	3 [2–4]	74	6 [3–8]
CCI-A, score	693	6 [5–8]	523	6 [5–7]	96	7 [5–8]	74	9 [7–12]
CIRS-G, score	693	11.8 ± 4.69	523	11.3 ± 4.57	96	12.6 ± 4.78	74	14.1 ± 4.57
CIRS-G severity index	693	1.91 ± 0.43	523	1.88 ± 0.42	96	1.93 ± 0.40	74	2.16 ± 0.43
Functional status								
ADL, score	673	2 [1–3]	510	2 [1–3]	90	1 [1–3]	73	1 [1–2]
IADL, score	673	1 [0–1]	510	1 [0–1]	90	1 [0–2]	73	1 [0–2]
Require walking aid	655	513 (78.3)	492	388 (78.9)	93	73 (78.5)	70	52 (74.3)
Cognitive impairment	693	441 (63.6)	523	343 (65.6)	96	60 (62.5)	74	38 (51.4)
Mortality	693	74 (10.7)	523	42 (8.0)	96	14 (14.6)	74	18 (24.3)
Time to death, days	693	122 [114–133]	523	122 [114–133]	96	122.5 [113–134]	74	119.5 [103–128]

All data were reported as mean ± SD, median [IQR], or *n* (%)

*CCI* Charlson Comorbidity Index, *CCI-A* Charlson Comorbidity Index age-adjusted, *CIRS-G* Cumulative Illness Rating Scale–Geriatric, *CIRS-G severity* total CIRS-G score divided by the total categories endorsed in CIRS-G, *ADL* activities of daily living, *IADL* instrumental activities of daily living, *SPPB* Short Performance Battery Score

The median CCI and CCI-A score were 2 [1–4] and 6 [5–8], respectively. The prevalence of each condition included in the CCI is shown in supplementary table 1 (online resource 1). The prevalence of dementia was significantly lower in the active cancer group compared to the past and no cancer groups and, by definition, any malignancy was more common in the past and active cancer groups relative to the no cancer group. The mean CIRS-G score was 11.8 ± 4.69 with a mean CIRS-G severity score of 1.91 ± 0.43. The number of patients with a score greater than zero, and the median score for each organ system is shown in supplementary table 2 (online resource 1), stratified by cancer status group. Patients with active cancer had a higher prevalence of hematological, hepatic/pancreatic, and genitourinary disorders compared to those with past or no cancer. Cancer type and stage are detailed in supplementary table 3 (online resource 1). The six most commonly occurring solid organ cancers were colorectal, prostate, breast, bladder, melanoma, and lung. The majority of patients with active cancer had metastatic or locally advanced disease, while patients with past cancer had early stage cancers. The six most common reasons for hospitalization are shown in supplementary table 4 (online resource 1). Geriatric rehabilitation inpatients with active cancer were more likely to be hospitalized due to infection, gastrointestinal disorders, and those classified as “other” compared to patients with no or past cancer.

**Fig. 1:**
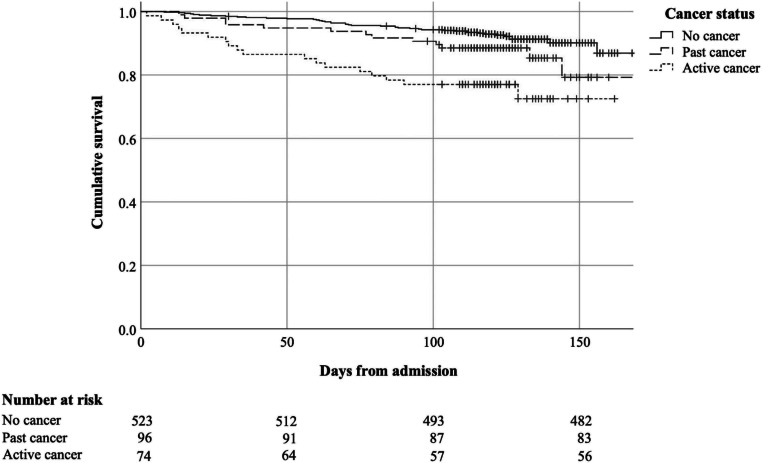
Kaplan–Meier survival curves of geriatric rehabilitation patients stratified by cancer status

Figure 1 illustrates 3-month survival by cancer status. At 3 months post discharge, the number of patients deceased in each cancer status group (no history, past, active) was 42 (8%), 14 (14.6%), and 18 (24.3%), respectively. After adjusting for age and sex, patients with active cancer and past cancer had a higher mortality risk compared to patients with no cancer (HR = 3.57, 95% CI 2.03–6.23 and HR = 1.78, 95% CI 0.97–3.28, respectively) (Table 2).

**Table 2:** The association of cancer status and all-cause mortality

Cancer status group	No cancer as reference				Past cancer as reference			
	Unadjusted		Adjusted		Unadjusted		Adjusted	
	HR (95% CI)	*p*	HR (95% CI)	*p*	HR (95% CI)	*p*	HR (95% CI)	*p*
No cancer	1.00	–	1.00	–	–	–	–	–
Past cancer	1.79 (0.98–3.28)	0.060	1.78 (0.97–3.28)	0.065	1.00	–	1.00	–
Active cancer	**3.60 (2.07–6.26)**	**< 0.001**	**3.57 (2.03–6.27)**	**< 0.001**	**2.07 (1.01–4.23)**	**0.047**	**2.13 (1.04–4.37)**	**0.040**

All adjusted Cox regression analysis was adjusted for age and sex unless stated otherwise

Table 3 shows the association between the multimorbidity measures and mortality, stratified by cancer status. CCI and CCI-A scores (per one-point increment) were significantly associated with a higher risk of mortality in the no cancer group (HR = 1.21, 95% CI 1.06–1.37; HR = 1.22, 95% CI 1.08–1.37), past cancer group (HR = 1.27, 95% CI 1.06–1.52; HR = 1.26, 95% CI 1.05–1.50), and active cancer group (HR = 1.16, 95% CI 1.00–1.34; HR = 1.15, 95% CI 1.00–1.33).

**Table 3:** The association of multimorbidity scores and all-cause mortality, stratified by cancer status

Multimorbidity scores	No cancer (*n* = 523)				Past cancer (*n* = 96)				Active cancer (*n* = 74)			
	Unadjusted		Adjusted		Unadjusted			Adjusted			Unadjusted	Adjusted
	HR (95% CI)	*p*	HR (95% CI)	*p*	HR (95% CI)	*p*	HR (95% CI)	*p*	HR (95% CI)	*p*	HR (95% CI)	*p*
CCI	1.21 (1.07–1.36)	0.002	1.21 (1.06–1.37)	0.003	1.25 (1.05–1.48)	0.012	1.27 (1.06–1.52)	0.009	1.15 (0.99–1.32)	0.054	1.16 (1.00–1.34)	0.044
CCI-Aa	1.23 (1.09–1.38)	< 0.001	1.22 (1.08–1.37)	0.001	1.23 (1.04–1.46)	0.017	1.26 (1.05–1.50)	0.012	1.14 (0.99–1.30)	0.062	1.15 (1.00–1.33)	0.046
CIRS-G	1.09 (1.02–1.16)	0.007	1.09 (1.02–1.16)	0.007	1.11 (0.99–1.23)	0.059	1.11 (0.99–1.23)	0.058	1.07 (0.97–1.18)	0.168	1.09 (0.98–1.20)	0.128
CIRS-G severity	1.62 (0.81–3.24)	0.169	1.77 (0.87–3.61)	0.115	0.95 (0.28–3.24)	0.935	0.96 (0.26–3.60)	0.956	1.24 (0.45–3.40)	0.678	1.44 (0.52–4.04)	0.486

All adjusted Cox regression analysis was adjusted for age and sex unless stated otherwise

*CCI* Charlson Comorbidity Index, *CCI-A* Charlson Comorbidity Index age-adjusted, *CIRS-G* Cumulative Illness Rating Scale–Geriatric, *CIRS-G severity* total CIRS-G score divided by the total categories endorsed in CIRS-G

^a^Cox regression analysis only adjusted for sex

CIRS-G score was associated with mortality in the no cancer group (HR = 1.09, 95% CI 1.02–1.16) but not associated in the past cancer (HR = 1.11, 95% CI 0.99–1.23) and active cancer groups (HR = 1.09, 95% CI 0.98–1.20). The CIRS-G severity index was not statistically significantly associated with mortality in any cancer status group (no cancer—HR = 1.77, 95% CI 0.87–3.61; past cancer—HR = 0.96, 95% CI 0.26–3.60; active cancer—HR = 1.44, 95% CI 0.52–4.04).

## Discussion

Incremental CCI and CCI-A scores were significantly associated with higher mortality, regardless of cancer status. CIRS-G scores were significantly associated with higher mortality in patients without cancer and displayed a trend toward increased mortality risk in patients with past cancer. The CIRS-G severity scores were not associated with higher mortality risk in any of the three cancer status groups. Geriatric rehabilitation inpatients with active cancer had significantly higher 3-month mortality compared to those with past or no cancer.

The CCI and CIRS are two of the most commonly utilized comorbidity indices [5] and have been related to mortality in community dwelling individuals, acute inpatients, and in patients with various cancers [17]. Admission to geriatric rehabilitation typically occurs following an episode of acute illness or trauma that is associated with a deterioration in an older patient’s functional and/or cognitive abilities that prevents their return home once medical stability has been achieved. Despite recognition that CGA contributes to the management of such patients, and that the assessment of comorbidities is an essential component of CGA [18], it has been previously identified that there is little published research in this area [19], and to the best of our knowledge, none examining the association between commonly utilized comorbidity scores and mortality according to cancer status.

By the age of 85 years, one in two older Australians will have survived or be living with cancer [20]. The prevalence of a cancer diagnosis in our cohort, almost 25%, was less than this, reflecting the selected nature of patients admitted to geriatric rehabilitation wards. The significantly higher mortality in those with active cancer, compared to those with past and no cancer history, was expected given the majority had incurable disease.

The median CCI scores in each of the three groups were higher than that reported in other studies in geriatric rehabilitation patients [21, 22]. The higher mortality with incremental CCI and CCI-A scores in all three cancer status groups demonstrates that the association of multimorbidity with mortality is independent of cancer status in geriatric rehabilitation patients, and that this simple tool contributes information about short-term mortality in this population. The prevalence of the individual diseases comprising the CCI did not differ between the three cancer status groups, with the exception of the malignancies and a lower number of dementia patients in the active cancer group. While lower rates of dementia have been documented in some cohorts of older cancer patients [23], the difference here is likely due to selection bias, with patients with both advanced cancer and dementia following a palliative rather than rehabilitative pathway. Taken together, these findings reflect that the cancer diagnosis, which in this cohort was largely metastatic or locally advanced in nature, determined the higher mortality in the active cancer group. For these patients, careful consideration of the role and goals of geriatric rehabilitation is essential. Many patients and families prefer to spend the last part of their life in the community, rather than a hospital, and a patient-centered approach that accounts for this is critical.

The mean CIRS-G score of our cohort was also higher than that reported in other studies of geriatric rehabilitation [24] and older cancer patients [6, 25]. The past and active cancer groups had a higher prevalence of genitourinary disorders, likely explained by the number of prostate and bladder cancers in these groups. The active cancer group had a higher prevalence of hepatic/pancreatic and hematological conditions. The latter is likely explained by the association of anemia with many advanced malignancies and the difficulties in rating hematological comorbidity in patients with cancer using the CIRS, which have been previously, explored [25]. Our finding that the CCI was more consistently associated with mortality is consistent with findings in other settings, such as patients with chronic disability [26], and with studies of older cancer patients demonstrating that the correlation between CCI and CIRS-G scores in older cancer patients is fair [27]. It has been previously documented that the two scales provide different “quantitative and qualitative” information regarding comorbidity [6, 27]. The lack of association between CIRS-G scores and mortality in the active cancer group is likely explained by the more comprehensive nature of the CIRS-G, which results in a number of minor conditions that are highly unlikely to influence the prognosis of a patient with metastatic or advanced cancer, contributing to the score, thus reducing its association with mortality in this group.

### Strengths and limitations

The major limitation of this study is the small number of patients of each cancer type within the past and active cancer groups. As a result, analysis of differences between cancer types was not possible, nor was it possible to explore the role of previous anti-cancer treatment(s), particularly in the past cancer group.

To the best of our knowledge, this is the largest prospective study to provide detailed information regarding the comorbidity profile of geriatric rehabilitation inpatients, and the first to examine the association between multimorbidity and mortality among different cancer status groups in this setting.

## Conclusion

While multimorbidity is associated with higher mortality in geriatric rehabilitation patients in all cancer status groups, those with active cancer have significantly higher 3-month mortality than those with no or past cancer. This is likely determined by the cancer diagnosis, often advanced, itself.

## Supplementary Information

ESM 1 (DOCX 50 kb)

## Data Availability

Data for this analysis include patients participating in the RESORT study between 15 October 2017 and 31 August 2018. Requests for access to this data can be made in writing to the corresponding author.

## References

[1] World Health Organization. Latest global cancer data: cancer burden rises to 18.1 million new cases and 9.6 million cancer deaths in 2018. GLOBOCAN2018

[2] American Cancer Society. Global Cancer Facts & Figures 4th Edition. Atlanta: American Cancer Society2018

[3] Salive ME (2013). Multimorbidity in older adults. Epidemiol Rev.

[4] Sogaard M, Thomsen RW, Bossen KS, Sorensen HT, Norgaard M (2013). The impact of comorbidity on cancer survival: a review. Clin Epidemiol.

[5] Soh CH, Ul Hassan SW, Sacre J, Maier AB (2020). Morbidity measures predicting mortality in inpatients: a systematic review. J Am Med Dir Assoc.

[6] Extermann M (2000). Measurement and impact of comorbidity in older cancer patients. Crit Rev Oncol Hematol.

[7] Read WL, Tierney RM, Page NC, Costas I, Govindan R, Spitznagel EL (2004). Differential prognostic impact of comorbidity. J Clin Oncol.

[8] Ellis G, Gardner M, Tsiachristas A, Langhorne P, Burke O, Harwood RH, Conroy SP, Kircher T, Somme D, Saltvedt I, Wald H, O'Neill D, Robinson D, Shepperd S, Cochrane Effective Practice and Organisation of Care Group (2017). Comprehensive geriatric assessment for older adults admitted to hospital. Cochrane Database Syst Rev.

[9] Folstein MF, Folstein SE, McHugh PR (1975). “Mini-mental state”: a practical method for grading the cognitive state of patients for the clinician. J Psychiatr Res.

[10] Nasreddine ZS, Phillips NA, Bédirian V, Charbonneau S, Whitehead V, Collin I (2005). The Montreal Cognitive Assessment, MoCA: a brief screening tool for mild cognitive impairment. J Am Geriatr Soc.

[11] Storey JE, Rowland JTJ, Basic D, Conforti DA, Dickson HG (2004). The Rowland Universal Dementia Assessment Scale (RUDAS): a multicultural cognitive assessment scale. Int Psychogeriatr.

[12] Katz S, Ford AB, Moskowitz RW, Jackson BA, Jaffe MW (1963). Studies of illness in the aged. The index of ADL: a standardized measure of biological and psychosocial function. JAMA..

[13] Lawton MP, Brody EM (1969). Assessment of older people: self-maintaining and instrumental activities of daily living. Gerontologist..

[14] Charlson ME, Pompei P, Ales KL, MacKenzie CR (1987). A new method of classifying prognostic comorbidity in longitudinal studies: development and validation. J Chronic Dis.

[15] Charlson M, Szatrowski TP, Peterson J, Gold J (1994). Validation of a combined comorbidity index. J Clin Epidemiol.

[16] Miller MD, Towers A (1991) A manual of guidelines for scoring the Cumulative Illness Rating Scale for Geriatrics (CIRS-G)

[17] Degroot V, Beckerman H, Lankhorst G, Bouter L (2003). How to measure comorbidity: a critical review of available methods. J Clin Epidemiol.

[18] Parker SG, McCue P, Phelps K, McCleod A, Arora S, Nockels K, Kennedy S, Roberts H, Conroy S (2018). What is comprehensive geriatric assessment (CGA)? An umbrella review. Age Ageing.

[19] Achterberg WP, Cameron ID, Bauer JM, Schols JM (2019). Geriatric rehabilitation—state of the art and future priorities. J Am Med Dir Assoc.

[20] Australian Institute of Health and Welfare. Cancer in Australia: In brief 2019. Canberra: AIHW2019. Cat. no. CAN 126

[21] Kabboord AD, Godfrey D, Gordon AL, Gladman JRF, Van Eijk M, van Balen R (2020). The modified functional comorbidity index performed better than the Charlson index and original functional comorbidity index in predicting functional outcome in geriatric rehabilitation: a prospective observational study. BMC Geriatr.

[22] Kabboord AD, Van Eijk M, Buijck BI, Koopmans R, van Balen R, Achterberg WP (2018). Comorbidity and intercurrent diseases in geriatric stroke rehabilitation: a multicentre observational study in skilled nursing facilities. Eur Geriatr Med.

[23] Zeber JE, Copeland LA, Hosek BJ, Karnad AB, Lawrence VA, Sanchez-Reilly SE (2008). Cancer rates, medical comorbidities, and treatment modalities in the oldest patients. Crit Rev Oncol Hematol.

[24] Guido D, Perna S, Peroni G, Guerriero F, Rondanelli M (2015). A comorbidity prognostic effect on post-hospitalization outcome in a geriatric rehabilitation setting: the pivotal role of functionality, assessed by mediation model, and association with the Brass index. Aging Clin Exp Res.

[25] Wedding U, Roehrig B, Klippstein A, Steiner P, Schaeffer T, Pientka L, Höffken K (2007). Comorbidity in patients with cancer: prevalence and severity measured by cumulative illness rating scale. Crit Rev Oncol Hematol.

[26] Rochon PA, Katz JN, Morrow LA, McGlinchey-Berroth R, Ahlquist MM, Sarkarati M (1996). Comorbid illness is associated with survival and length of hospital stay in patients with chronic disability. A prospective comparison of three comorbidity indices. Med Care.

[27] Extermann M, Overcash J, Lyman GH, Parr J, Balducci L (1998). Comorbidity and functional status are independent in older cancer patients. J Clin Oncol.

