# Supplementary material for: The association of comorbidity measures and mortality in geriatric rehabilitation inpatients by cancer status: RESORT
Source: Support Care Cancer. 2021 Jan 18;29(8):4513–9. doi: 10.1007/s00520-020-05967-z (PMC8236474; doi:10.1007/s00520-020-05967-z)
Supplement: Supplementary file 1 — (DOCX 50 kb) [file 520_2020_5967_MOESM1_ESM.docx]

**Supplementary Table 1.** Charlson comorbidity index and prevalence of component diseases, stratified by cancer status

| **Index score** | **Condition** | **No cancer**  **(n = 523)** | |  | **Past cancer**  **(n = 96)** | |  | **Active cancer**  **(n = 74)** | |
| --- | --- | --- | --- | --- | --- | --- | --- | --- | --- |
|  |  | **n** | **%** |  | **n** | **%** |  | **n** | **%** |
| 1 | Myocardial infarct | 109 | 20.8 |  | 26 | 27.1 |  | 14 | 18.9 |
|  | Congestive heart failure | 111 | 21.2 |  | 23 | 24.0 |  | 14 | 18.9 |
|  | Peripheral vascular disease | 30 | 5.7 |  | 7 | 7.3 |  | 6 | 8.1 |
|  | Cerebrovascular disease (not hemiplegia) | 123 | 23.5 |  | 23 | 24.0 |  | 14 | 18.9 |
|  | Dementia* | 118 | 22.6 |  | 21 | 21.9 |  | 6 | 8.1 |
|  | Chronic pulmonary disease | 98 | 18.7 |  | 17 | 17.7 |  | 11 | 14.9 |
|  | Connective tissue disease | 30 | 5.7 |  | 3 | 3.1 |  | 3 | 4.1 |
|  | Peptic ulcer disease | 44 | 8.4 |  | 6 | 6.3 |  | 3 | 4.1 |
|  | Mild liver disease | 27 | 5.2 |  | 6 | 6.3 |  | 7 | 9.5 |
|  | Diabetes without end organ damage | 100 | 19.1 |  | 16 | 16.7 |  | 13 | 17.6 |
| 2 | Diabetes with end organ damage | 86 | 16.4 |  | 14 | 14.6 |  | 15 | 20.3 |
|  | Hemiplegia | 24 | 4.6 |  | 3 | 3.1 |  | 4 | 5.4 |
|  | Moderate to severe renal disease | 98 | 18.7 |  | 21 | 21.9 |  | 15 | 20.3 |
|  | Any non-metastatic tumour* | 8 | 1.5 |  | 22 | 22.9 |  | 28 | 37.8 |
|  | Leukaemia* | 1 | 0.2 |  | 1 | 1.0 |  | 14 | 18.9 |
|  | Malignant lymphoma* | 0 | 0.0 |  | 0 | 0.0 |  | 3 | 4.1 |
| 3 | Moderate or severe liver disease | 9 | 1.7 |  | 1 | 1.0 |  | 3 | 4.1 |
| 6 | Metastatic solid tumour* | 0 | 0.0 |  | 2 | 2.1 |  | 31 | 40.5 |
|  | AIDS or AIDS related complex (not just HIV) | 1 | 0.2 |  | 0 | 0.0 |  | 0 | 0.0 |

** p* < 0.05 in Chi-square test or Fishers exact test

**Supplementary Table 2.** Cumulative illness rating scale- geriatric version: prevalence of disorder within and median score for each organ system, stratified by cancer status

| **Organ system** | **No cancer**  **(n = 523)** | |  | **Past cancer**  **(n = 96)** | |  | **Active cancer**  **(n = 74)** | |
| --- | --- | --- | --- | --- | --- | --- | --- | --- |
|  | **n≥1** | **Median** |  | **n≥1** | **Median** |  | **n≥1** | **Median** |
| Cardiac | 335 | 1 [0-2] |  | 58 | 1 [0-2] |  | 40 | 1 [0-2] |
| Vascular | 347 | 1 [0-2] |  | 56 | 1 [0-2] |  | 45 | 1 [0-1] |
| Haematological* | 120 | 0 [0-0] |  | 21 | 0 [0-0] |  | 38 | 1 [0-2] |
| Respiratory | 189 | 0 [0-1] |  | 40 | 0 [0-1.5] |  | 33 | 0 [0-2] |
| Ophthalmological and ORL | 167 | 0 [0-1] |  | 38 | 0 [0-1] |  | 25 | 0 [0-1] |
| Upper gastrointestinal | 163 | 0 [0-1] |  | 37 | 0 [0-1] |  | 29 | 0 [0-1] |
| Lower gastrointestinal* | 171 | 0 [0-1] |  | 53 | 1 [0-2] |  | 36 | 0 [0-2] |
| Hepatic and pancreatic* | 72 | 0 [0-0] |  | 17 | 0 [0-0] |  | 20 | 0 [0-1] |
| Renal | 171 | 0 [0-1] |  | 36 | 0 [0-1.5] |  | 27 | 0 [0-2] |
| Genitourinary* | 183 | 0 [0-1] |  | 48 | 0.5 [0-2] |  | 41 | 1 [0-3] |
| Musculoskeletal and integumental | 380 | 2 [0-2] |  | 66 | 2 [0-3] |  | 47 | 1 [0-2] |
| Neurological | 231 | 0 [0-2] |  | 44 | 0 [0-2] |  | 34 | 0 [0-2] |
| Endocrine/metabolic and breast | 353 | 1 [0-2] |  | 70 | 1 [0-2] |  | 52 | 1.5 [0-2] |
| Psychiatric | 269 | 1 [0-2] |  | 43 | 0 [0-2] |  | 31 | 0 [0-2] |

All data were reported as median [IQR]. ** p* < 0.05 in both Chi-square test and Kruskal-Wallis test.

**Supplementary Table 3.** Cancer type and stage of patients, stratified by cancer status

|  | **Past cancer**  **(n = 96)** | |  | **Active cancer**  **(n = 74)** | |
| --- | --- | --- | --- | --- | --- |
|  | **n** | **%** |  | **n** | **%** |
| **Cancer type**^a^ |  |  |  |  |  |
| Colorectal | 31 | 32.3 |  | 9 | 12.2 |
| Prostate | 15 | 15.6 |  | 17 | 23.0 |
| Breast | 15 | 15.6 |  | 4 | 5.4 |
| Bladder | 9 | 9.4 |  | 5 | 6.8 |
| Melanoma | 8 | 8.3 |  | 4 | 5.4 |
| Lung | 3 | 3.1 |  | 5 | 6.8 |
| Kidney | 4 | 4.2 |  | 3 | 4.1 |
| Liver | 0 | 0.0 |  | 3 | 4.1 |
| Leukaemia | 0 | 0.0 |  | 11 | 14.9 |
| Lymphoma | 1 | 1.0 |  | 4 | 5.4 |
| Multiple Myeloma | 0 | 0.0 |  | 1 | 1.4 |
| Laryngeal | 2 | 2.1 |  | 1 | 1.4 |
| Thyroid | 2 | 2.1 |  | 0 | 0.0 |
| Other* | 15 | 15.6 |  | 18 | 24.3 |
| **Cancer stage**^b^ |  |  |  |  |  |
| Early | 77 | 80.2 |  | 13 | 17.6 |
| Late | 4 | 4.2 |  | 30 | 40.5 |
| Unknown | 15 | 15.6 |  | 31 | 41.9 |

^*^ Other cancer includes anal, cervical, spindle cell carcinoma, cholangiocarcinoma, endometrial, gallbladder, gastric, head and neck, ovarian, pancreatic, parotid, thymoma, tongue squamous cell carcinoma and uterine. ^a^ Cancer type does not add up to the total number of cancer patients as patients could have multiple cancer types. ^b^ The overall cancer stage was determined by the stage of the predominant cancer.

**Supplementary Table 4.** Primary reason for hospitalisation, stratified by cancer status

|  | **No cancer**  **(n = 523)** | |  | **Past cancer**  **(n = 96)** | |  | **Active cancer**  **(n = 74)** | |
| --- | --- | --- | --- | --- | --- | --- | --- | --- |
|  | **n** | **%** |  | **n** | **%** |  | **n** | **%** |
| Musculoskeletal* | 257 | 49.1 |  | 44 | 45.8 |  | 24 | 32.4 |
| Neurological | 94 | 18.0 |  | 15 | 15.6 |  | 10 | 13.5 |
| Infection* | 55 | 10.5 |  | 16 | 16.7 |  | 14 | 18.9 |
| Cardiaovascular | 47 | 9.0 |  | 7 | 7.3 |  | 3 | 4.1 |
| Gastrointestinal* | 30 | 5.7 |  | 7 | 7.3 |  | 11 | 14.9 |
| Respiratory | 24 | 4.6 |  | 5 | 5.2 |  | 2 | 2.7 |
| Other^a^* | 16 | 3.1 |  | 2 | 2.1 |  | 10 | 13.5 |

^a^ Other primary reason for hospitalisation includes urology, metabolic, psychiatric, vascular, haematologic and ophthalmological. ** p* < 0.05 in Chi-square test or Fishers exact test.
